# A systematic review on neutrophils interactions with titanium and zirconia surfaces: Evidence from in vitro studies

**DOI:** 10.1002/cre2.582

**Published:** 2022-05-10

**Authors:** Gayathiri Elangovan, Joao M. Mello‐Neto, Santosh K. Tadakamadla, Peter Reher, Carlos Marcelo S. Figueredo

**Affiliations:** ^1^ School of Medicine and Dentistry Griffith University Southport Queensland Australia; ^2^ Affiliated to research, Department of Dental Medicine Karolinska Institutet Solna Sweden

**Keywords:** neutrophils, systematic review, titanium, zirconia

## Abstract

**Objectives:**

This systematic review aimed to assess in vitro studies that evaluated neutrophil interactions with different roughness levels in titanium and zirconia implant surfaces.

**Material and Methods:**

An electronic search for literature was conducted on PubMed, Embase, Scopus, and Web of Science and a total of 14 studies were included. Neutrophil responses were assessed based on adhesion, cell number, surface coverage, cell structure, cytokine secretion, reactive oxygen species (ROS) production, neutrophil activation, receptor expression, and neutrophil extracellular traps (NETs) release. The method of assessing the risk of bias was done using the toxicological data reliability assessment tool (TOXRTOOL).

**Results:**

Ten studies have identified a significant increase in neutrophil functions, such as surface coverage, cell adhesion, ROS production, and NETs released when interacting with rough titanium surfaces. Moreover, neutrophil interaction with rough–hydrophilic surfaces seems to produce less proinflammatory cytokines and ROS when compared to naive smooth and rough titanium surfaces. Regarding membrane receptor expression, two studies have reported that the FcγIII receptor (CD16) is responsible for initial neutrophil adhesion to hydrophilic titanium surfaces. Only one study compared neutrophil interaction with titanium alloy and zirconia toughened alumina surfaces and reported no significant differences in neutrophil cell count, activation, receptor expression, and death.

**Conclusions:**

There are not enough studies to conclude neutrophil interactions with titanium and zirconia surfaces. However, different topographic modifications such as roughness and hydrophilicity might influence neutrophil interactions with titanium implant surfaces.

## 1 INTRODUCTION

Ever since the ground‐breaking work of Brånemark in the 1960s, different metals and their alloys have been used as dental implants due to their excellent biocompatibility, mechanical strength, and aesthetics (Brånemark et al., 1969). When an implant is inserted into the bone, an initial inflammatory response is triggered by the immune cells of myeloid origin predominated by neutrophils (Kolaczkowska & Kubes, 2013; Segal, 2005). Neutrophils, the critical cellular player, activate an inflammatory cascade by producing cytokines, enzymes, and DNA fiber networks called neutrophil extracellular traps (NETs) (Brinkmann et al., 2004; Nauseef, 2016). The presumed neutrophil response to a dental implant is determined by several physical and chemical features of the implant surfaces, which include mechanical and physicochemical properties such as chemical composition, surface wettability, surface energy, and surface topography (Bowers et al., 1992; Galli et al., 2005; Ong et al., 1996).

Improvements in osteointegration have been achieved by introducing micro and nano roughness on the implant surfaces (Goené et al., 2007; Grandfield et al., 2013; Jarmar et al., 2008). Titanium implant surfaces were classified according to the degree of roughness into four categories: smooth (Sa = 0.0–0.4 µm), minimally rough (Sa = 0.5–1.0 µm), moderately rough (Sa = 1.0–2.0 µm), and rough (Sa > 2.0 µm) (Wennerberg & Albrektsson, 2009). Based on this classification, studies have demonstrated stronger/increased adhesion of neutrophils on rough titanium implant surfaces than on smooth Ti surfaces (Campos et al., 2014; Vitkov et al., 2015). Studies have also identified different neutrophil morphology and NETotic responses to titanium surfaces with various roughness fields (Abaricia et al., 2020; Vitkov et al., 2015). Furthermore, researchers have investigated neutrophil interaction with implant surfaces having a combination of roughness/hydrophilicity, which may promote quicker healing time and reduced initial inflammatory response (Abaricia et al., 2020; El Kholy et al., 2020).

Zirconia is considered a potential alternative to titanium implants due to its aesthetics, excellent biocompatibility, mechanical properties, and reduced bacterial biofilm formation (Christel et al., 1989; Langhoff et al., 2008; Piconi & Maccauro, 1999). However, due to its high hardness, surface roughening of zirconia has been technically challenging (Rottmar et al., 2019). Different surface topographies of zirconia were reported to increase osteoblast proliferation on a rough surface compared to a smooth surface (Bächle et al., 2007). A recent study showed a significantly increased cellular spreading and migration rate on rough zirconia surfaces (Sa = 3.36 μm) than on the rough titanium implant surfaces (Munro et al., 2020). From these studies mentioned above, it is assumed that the functional activity of neutrophils is determined by the implant surface characteristics raising questions regarding the exact nature of such interactions. Therefore, this systematic review aimed to assess in vitro studies that evaluated neutrophil interactions with different roughness levels in titanium and zirconia implant surfaces.

## 2 MATERIALS AND METHODS

The reporting of this review complies with the Preferred Reporting Items for Systematic Reviews and Meta‐Analyses (PRISMA) statement guidelines (Moher et al., 2009, 2012). The PRISMA checklist is presented in Supporting Information Materials S1 and S2. Ethics approval was not required for this systematic review.

### 2.1 Literature search strategy

Two independent reviewers (G. E. and J. M. M.‐N.) conducted an electronic search done up to April 2021, using Medical Subject Headings, keywords, and other accessible terms on PubMed. The search strategy was adapted to other electronic databases, including Embase, Scopus, and Web of Science. Appropriate Boolean operators (OR, AND) were used to refine the searches. The search strategy used in PubMed was: (titanium OR zirconia) AND (neutrophils OR phagocyte OR neutrophils OR leukocyte OR granulocyte). No date or language limitations were placed during the search. The search strategy of the other databases is presented in Supporting Information Material S3.

All the retrieved titles and abstracts were exported to a referencing software program (EndNote X9, Philadelphia, Clarivate). Any duplicates found were deleted. Two reviewers (G. E. and J. M. M.‐N.) screened all the titles and abstracts independently; those that seemed suitable were considered for inclusion in the full‐text review. When the information provided in the abstract and title were inadequate to determine eligibility, articles were reviewed in full. Disagreement between reviewers (G. E. and J. M. M.‐N.) was resolved through discussion. A third reviewer (C. M. S. F.) was consulted when an agreement was not reached.

### 2.2 Eligibility criteria

The eligibility criteria were based on the PICO (population, intervention, control, and outcomes) questions: How do neutrophils interact with titanium and zirconia surfaces? Studies conducted in vitro investigated peripheral neutrophils (leukocytes, polymorphonuclear (PMN) cells, granulocytes) on titanium and zirconia surfaces with or without surface topography modifications and articles in English. Studies were excluded in vivo and ex vivo investigating titanium nanoparticles, coated implant surfaces, and abstract studies.

### 2.3 Data extraction

Relevant data were extracted by two reviewers (G. E. and J. M. M.‐N.) independently. Authors were contacted if any missing data or additional data were required from the eligible studies. The following data items were extracted from the eligible studies: author (year); type of biomaterials used in the test and comparison groups; experimental design (methods); primary outcomes (results) related to neutrophils morphology, NETs release, cytokine secretion, neutrophils adhesion, neutrophils receptor expression, ROS production and phagocytosis ability of neutrophils on titanium and zirconia surfaces. A narrative synthesis of the findings from the included studies is presented based on neutrophil response to roughness, hydrophilicity, cytokine expression, morphology, NETosis, and receptor expression (Table 1).

**Table 1:** Characteristics of the included studies

Author and year	Material/surface modification	Cells	Methods	Main finding
Abaricia et al. (2020)	Ti	Neutrophils isolated from murine blood	Surface analysis; flow cytometry; coculture; SEM and CLSM; ELISA; qPCR	– Neutrophils on rough‐hydro Ti surfaces released decreased levels of IL‐1β, IL‐6, IL‐12, TNF‐α, IL, neutrophil elastase, and MPO, as well as decreased NET formation compared to smooth and rough Ti surfaces.
	– Smooth – Rough – Rough‐hydrophilic			
Radley et al. (2019)	Highly polished	Human peripheral blood	Flow cytometry; ELISA; phagocytosis; ROS assay; rheometry	The application of shear stress to blood on Ti alloy surfaces leads to neutrophil activation indicated by reduced l‐selectin expression.
	– Diamond‐like carbon‐coated stainless steel – Single‐crystal sapphire – Ti alloy			
Radley et al. (2018)	Highly polished diamond‐like carbon‐coated stainless steel	Human peripheral blood	Flow cytometry; ELISA	– No significant neutrophil response was found on Ti and zirconia surfaces.
	– Single‐crystal sapphire – Zirconia‐toughened alumina – Ti alloy			
Vitkov et al. (2015)	Ti	Human peripheral blood	SEM; CLSM; immunocytochemistry	– Human neutrophils rapidly adhered to SLA surfaces, triggering histone citrullination, and NET release. – Albumin or acetylsalicylic acid had no significant effects on the inflammatory response to SLA surfaces.
	– SLA surface – SLA coated with albumin – SLA coated with albumin/acetylsalicylic acid			
Campos et al. (2014)	Ti	Neutrophils isolated from human blood	SEM; flowcytometry; AFM	– The adhesion of neutrophils to the “rough” Ti surface was initially stronger than adhesion to the “smooth” surface. – Neutrophils adhering to the rough surface had a fourfold higher surface attachment. – Cells adhering to the rough surfaces showed prominent shape changes and more cytoplasmic surface projection. – Expression of l‐selectin and CD11b were influenced by neutrophils but not by different Ti surface structures.
	– Smooth – rough			
Smith et al. (2013)	Titania nanotube and Ti surfaces	Human peripheral blood	AFM; MTT assay; ELISA	– Short‐ and long‐term exposure of neutrophils on rough Ti surfaces showed increased adhesion, and proliferation when compared to nanostructural surfaces.
Arvidsson et al. (2011)	Ti blasted with Al_2_O_3_	Human peripheral blood	Respiratory burst; SEM	– Viable cell count and ROS production had no significant difference between the Ti surfaces investigated.
	– Alkali and heat treated – Fluoride treated – HA coating			
Schildhauer et al. (2009)	– Pure Ti – Ti alloy – Grit‐blasted stainless steel – Pure tantalum – Tantalum‐coated stainless steel – Porous tantalum foam material	Human peripheral blood	ELISA; SEM; chemotaxis, flow cytometry	– Activated neutrophils on smooth Ti and alloy surfaces released relatively low levels of IL‐ra (~80 pg/ml; ~120 pg/ml) and IL‐8 (~200 pg/ml; ~100 pg/ml).
Erikkson et al. (2001)	Ti sheets	Human peripheral blood	Immunofluorescence; chemiluminescence activity	– Neutrophils adhere to Ti in an Fc receptor‐dependent manner.
Erikkson et al. (2001)	Ti sheets	Human peripheral blood	Chemiluminescence; immunofluorescence; cell number	The present study results indicate that PMNLs recognize hydrophilic and hydrophobic Ti surfaces by different adhesion receptors and show different patterns of receptor expression.
Erikkson et al. (2001)	Ti sheets	Human peripheral blood	Surface analysis; viability staining; chemiluminescence; immunofluorescence	– The rough surfaces elicited a stronger biological response than the smooth and the surfaces with thick oxides had a dampening effect on most of the cellular reactions investigated. – Adhering leukocytes were susceptible to both changes in topography and composition of the TiO_2_ surfaces.
	– Smooth surface with thin TiO_2_ – Smooth surface with thick TiO_2_ – rough surface with thin TiO_2_ – Rough surface with thick TiO_2_			
Nygren et al. (1997)	Ti sheets	Human peripheral blood	Optical profilometry; SEM; Auger electron spectroscopy; X‐ray photoelectron; spectroscopy; immunofluorescence	– Priming of neutrophils (CD11b) was significantly higher on the rough Ti surfaces.
	– Annealed at 700°C – Immersed in 10% hydrofluoric acid			
Wilke et al. (1998)	– Hydroxyapatite ceramic, – Pure Ti – Ultra‐high‐molecular‐weight polyethylene	Human bone marrow cells	SEM; flow cytometry, fluorescence microscopy	–12.2 ± 2.4% of granulocytes (CD15‐positive cells) adhered to naive Ti surfaces.

Abbreviations: AFM, atomic force microscopy; CLSM, confocal laser scanning microscopy; ELISA, enzyme‐linked immunosorbent assay; MPO, myeloperoxidase; NET, neutrophil extracellular trap; PMNL, polymorphonuclear leukocyte; qPCR, quantitative polymerase chain reaction; SEM, scanning electron microscopy; TNF‐α, tumor necrosis factor‐α.

### 2.4 Risk of bias in the included studies

The method of assessing the risk of bias was done using the toxicological data reliability assessment tool (TOXRTOOL) (Schneider et al., 2009). For in vitro studies, it uses a set of 18 questions or criteria. For each criterion, a score of “1” is provided when the response is “yes,” or the criteria are addressed, while a score of “0” is given when the response is “no,” that is, when the criteria are not addressed in the study. If the value is ≥15, Category 1 is assigned. For values >11, Category 2 is assigned, and for all values <11, Category 3 is assigned. Categories 1 and 2 represent that the data is reliable without and with restriction, respectively, while Category 3 indicates that the data reported from the study is unreliable.

## 3 RESULTS

**Figure 1:**
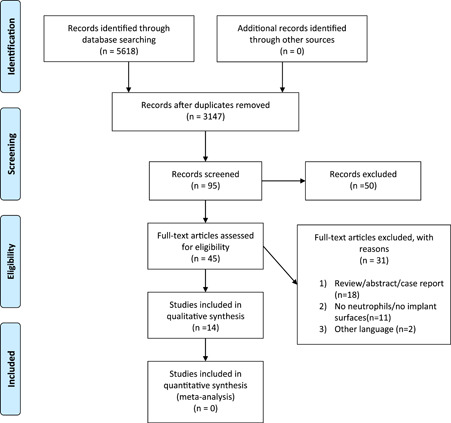
PRISMA flowchart of the online databases searched and selection of studies for inclusion.

The electronic search of the databases identified a total of 3147 papers: PubMed (*n* = 1110), Embase (*n* = 1090), Scopus (*n* = 666), and Web of Science (*n* = 281). After eliminating the duplicates and screening the titles and abstracts, 45 full texts were reviewed (Figure 1). Finally, 14 articles were included in the qualitative analysis. Table S1 depicts the studies excluded after full‐text review. The *κ*‐statistic for agreement on including full‐text articles between the reviewers was 1.0, indicating no disagreement. Table 1 represents the general characteristics of the selected studies.

### 3.1 Background characteristics of the included studies

Among the 14 selected studies, 10 articles compared neutrophils interactions with titanium surfaces of different roughness levels. Four articles compared neutrophil behaviors in titanium surfaces with other biomaterial surfaces used for medical implants, and among these, only one study compared neutrophil behavior between titanium and zirconia surfaces. All included studies assessed responses of peripheral neutrophils on titanium and zirconia surfaces (Abaricia et al., 2020; Arvidsson et al., 2011; Campos et al., 2014; El Kholy et al., 2020; Eriksson & Nygren, 2001a, 2001b; Eriksson et al., 2001; Nygren et al., 1997; Radley et al., 2018, 2019; Schildhauer et al., 2009; Smith et al., 2013; Vitkov et al., 2015; Wilke et al., 1998). Neutrophil responsiveness, morphological changes, and adhesion were evaluated about roughness in 10 studies. Different methods, such as 5 studies—enzyme‐linked immunosorbent assay, 9—scanning electron microscopy (SEM), 6—flowcytometry, 6—immunofluorescence staining, 5—chemiluminescence assay, 2—atomic force microscopy and 3‐(4,5‐dimethylthiazol‐2‐yl)‐2,5‐diphenyl tetrazolium bromide assay, 2—confocal laser scanning microscopy, were used to evaluate the role of cellular immune response and morphological changes on titanium and zirconia surfaces. Analysis of the geographic distribution revealed that five studies were carried out in Sweden (Arvidsson et al., 2011; Eriksson & Nygren, 2001a, 2001b; Eriksson et al., 2001; Nygren et al., 1997), three studies were carried out in the United States (Abaricia et al., 2020; El Kholy et al., 2020; Smith et al., 2013), two studies each in United Kingdom and Germany (Radley et al., 2018, 2019; Schildhauer et al., 2009; Wilke et al., 1998) and one study in Brazil (Campos et al., 2014). Five studies used neutrophils directly isolated from the blood, eight used whole blood (leukocytes/granulocytes/PMN), and one used human bone marrow cells (granulocytes). Table 1 summarizes the studies that evaluated immunological response, morphological changes, activation, and adhesion of neutrophils during interaction with titanium and zirconia implant surfaces.

### 3.2 Risk of bias

In this review, 12 studies were found to have an overall score of ≥15 (Category 1), demonstrating that data from these studies are reliable without restrictions. One study had a score of 14 and belonged to Category 2, and one study had a score of 11 and belonged to Category 3. The scoring for all studies is shown in Table S2 and Figure S1 shows the number of studies belonging to the respective categories.

### 3.3 Results from the individual studies

#### 3.3.1 Neutrophil behavior based on roughness

Ten studies assessed the interaction of neutrophils with titanium surfaces based on their roughness. One study identified intact cellular morphology and reduced chemiluminescence activity (respiratory burst) by neutrophils when interacting with serum coated moderately rough hydrophilic titanium surfaces compared to moderately rough hydrophobic titanium surfaces (El Kholy et al., 2020). A second study identified neutrophils interacting with rough hydrophilic titanium surfaces to produce low levels of interleukin‐1β (IL‐1β), IL‐6, IL‐12, tumor necrosis factor‐α, IL, neutrophils elastase, and myeloperoxidase with no NETs formation compared to neutrophils interacting with naive smooth and rough titanium surfaces (Abaricia et al., 2020). However, activated neutrophils on smooth titanium and alloy surfaces released relatively low IL‐ra and IL‐8 fields (Schildhauer et al., 2009). Additionally, rapid neutrophil adhesion (80%–82%) and various stages of NETosis with completely spread NETs with swollen nuclei and chromatin alteration on SLA (Sandblasted, Large grit, Acid‐etched) titanium surfaces were observed. (Vitkov et al., 2015). In another study, neutrophils exposed to rough titanium surfaces had a fourfold higher surface attachment area showing a prominent shape and more cytoplasmic projections after 2 h compared to smooth titanium surfaces (Campos et al., 2014). However, CD11b and l‐selectin expression in neutrophils were not influenced by titanium surface textures (Campos et al., 2014). Two studies also reported increased neutrophils adhesion, priming, ROS production and expression of CD 11b on rough titanium surfaces compared to smooth titanium surfaces (Eriksson et al., 2001; Nygren et al., 1997). Significant production of ROS occurred earlier on smooth titanium surfaces compared to rough titanium surfaces (Eriksson et al., 2001). No statistically significant differences in neutrophil cell count and production of ROS were observed on blasted Ti surfaces compared to other coated Ti surfaces (Arvidsson et al., 2011). Short‐ and long‐term exposure of neutrophils to rough titanium surfaces showed increased adhesion and proliferation compared to nanostructural surfaces (Smith et al., 2013). Only one study investigated biocompatibility parameters of human bone marrow cells and reported that 12.2% of granulocytes adhered to naive titanium surfaces (Wilke et al., 1998). Based on these results, increased neutrophil adhesion, ROS production, and different stages of NETosis were observed on rough titanium surfaces. However, rough hydrophilic titanium surfaces seem to induce decreased levels of proinflammatory cytokines and ROS production and showed no NET formation from neutrophils.

#### 3.3.2 Neutrophil behavior based on receptor expression

Eriksson and Nygren (2001b) investigated neutrophil functions based on adhesion receptors on hydrophilic and hydrophobic titanium surfaces. Eriksson and Nygren (2001a) found that neutrophils adhered to hydrophilic titanium surfaces in a FcγIII receptor (CD16). Expression of the FcγIII receptor on neutrophils was dominant during the initial hours, which gradually shifted towards CD11b expression later. Eriksson and Nygren (2001b) reported that neutrophil activation increased over time on hydrophilic titanium surfaces, which was evident from the decreased expression of CD62L. Additionally, the CD16 expression was higher during the initial hours at hydrophilic surfaces but only peaked after late hours at hydrophobic surfaces (Eriksson & Nygren, 2001b). The same study showed that neutrophils adhesion to hydrophilic and hydrophobic titanium surfaces was depressed by inhibiting hirudin (thrombin inhibition), reporting the expression of CD16 and CD11b to be thrombin dependent (Eriksson & Nygren, 2001b). A recent study also identified a significant reduction in l‐selectin (CD62L) expression on titanium alloy surfaces by applying sheave *r* force, indicating an increased neutrophils activation compared to other highly polished medical implant surfaces such as stainless steel and sapphire crystal (Radley et al., 2019). These studies indicate that different adhesion receptors recognize hydrophilic and hydrophobic titanium surfaces. Moreover, the activation and adhesion were increased in hydrophilic surfaces compared to hydrophobic surfaces.

#### 3.3.3 Neutrophil interaction with titanium and zirconia implant surfaces

Only one study compared neutrophils interaction between titanium alloy and zirconia toughened alumina surfaces (Radley et al., 2018). The neutrophils count was not significantly different between titanium and zirconia surfaces, and the neutrophil expression of CD62L did not differ between these surfaces.

## 4 DISCUSSION

This systematic review indicated that neutrophils functions, such as adhesion, surface coverage, attachment, and NET release, are influenced by topographic modifications on titanium surfaces. It has been demonstrated that a rapid surface coverage and stronger neutrophil adhesion can be seen on rough titanium surfaces compared to smooth titanium surfaces (Campos et al., 2014; Eriksson et al., 2001; Vitkov et al., 2015). Additionally, SEM analysis has shown different morphological features of neutrophils, such as flat cells, cytoplasmic projections, and surface attachment on rough titanium surfaces (Campos et al., 2014). Like neutrophils, macrophage adhesion, morphology, and phenotype can be modulated by implant surface roughness (Chen et al., 2010; Geiger et al., 2009; Soskolne et al., 2002). Also, previous studies have shown increased osteoblast adhesion on rough titanium surfaces compared to smooth one's Fields (Bowers et al., 1992; Michaels et al., 1991). Additionally, studies have shown that osteoblast morphology can vary between rough and smooth titanium surfaces (Bowers et al., 1992; Boyan et al., 2003). Therefore, early biological events such as cell behavior and functions seem to be influenced by surface roughness.

Neutrophils seem to produce low levels of proinflammatory cytokines and enzymes and high anti‐inflammatory cytokines when interacting with rough hydrophilic Ti surfaces (Abaricia et al., 2020). Likewise, studies on macrophages also showed low levels of proinflammatory cytokines in response to micro‐rough hydrophilic titanium surfaces (Alfarsi et al., 2014; Hamlet et al., 2012). Studies on osteoblast have also found that increased hydrophilic surfaces improved osteogenic differentiation (Olivares‐Navarrete et al., 2011; Vlacic‐Zischke et al., 2011). In addition, micro‐rough hydrophilic surfaces seem to be positively involved in the earlier onset of the osseointegration (Lang et al., 2011). These findings suggest that surface hydrophilicity seems to attenuate the production of proinflammatory cytokines and may promote faster wound healing. (Vitkov et al., 2015) showed that rough Ti surfaces triggered the NET release and histone citrullination. In contrast, Abaricia et al. (2020) demonstrated no NET formation on rough hydrophilic Ti surfaces compared to naive rough and smooth Ti surfaces. Neutrophils are generally known to release NETs upon activation by microbes, often dependent on ROS generation (Kolaczkowska & Kubes, 2013). However, studies have shown NET formation even under sterile conditions (Abaricia et al., 2020; Vitkov et al., 2015). Therefore, it is plausible to believe that implant surface roughness/chemistry can affect the NETotic response.

It has been shown that neutrophils interaction with rough Ti surfaces can induce ROS to release (Eriksson et al., 2001; El Kholy et al., 2020), which might lead to local tissue damage, delayed wound healing and even loosening of implants (Hwang et al., 2019; Segal, 2005). Studies have also reported that Ti ions released from implant surfaces trigger macrophages and osteoblast to produce increased ROS levels (Vermes et al., 2001; Żukowski et al., 2018). It is believed that hydrophilic surfaces can downregulate neutrophil activation, causing a decrease in ROS production (El Kholy et al., 2020). Thus, the initial inflammatory response by neutrophils seems to be altered by combining surface topography/hydrophilicity.

Our findings must be interpreted with caution. First, only one study compared neutrophils interaction between titanium alloy and alumina toughened zirconia implant surfaces showing no significant difference. Second, only in vitro studies were assessed. Thus, further clinical and in vivo studies are needed to confirm the relevance of in vitro findings. Finally, the lack of homogeneous quantitative data for meta‐analysis and methodological heterogeneity to assess the interaction/behavior of neutrophils can also be a drawback of the present systematic review.

## 5 CONCLUSION

There are not enough studies to draw any conclusion about neutrophil interactions with titanium and zirconia surfaces. However, different topographic modifications such as roughness and hydrophilicity might influence neutrophil interactions with titanium implant surfaces.

## AUTHOR CONTRIBUTIONS

*Conceptualization*: Carlos Marcelo S. Figueredo. *Methodology*: Gayathiri Elangovan, Joao M. Mello‐Neto, Santosh K. Tadakamadla, and Carlos Marcelo S. Figueredo. *Validation*: Gayathiri Elangovan and Joao M. Mello‐Neto. *Formal analysis*: Gayathiri Elangovan and Joao M. Mello‐Neto. *Investigation*: Gayathiri Elangovan and Joao M. Mello‐Neto. *Resources*: Gayathiri Elangovan and Joao M. Mello‐Neto. *Data curation*: Gayathiri Elangovan and Joao M. Mello‐Neto. *Writing—original draft preparation*: Gayathiri Elangovan and Joao M. Mello‐Neto. *Writing, review, and editing*: Carlos Marcelo S. Figueredo, Gayathiri Elangovan, Joao M. Mello‐Neto, Peter Reher, and Santosh K. Tadakamadla. *Visualization*: Carlos Marcelo S. Figueredo, Gayathiri Elangovan, Joao M. Mello‐Neto, Peter Reher, and Santosh K. Tadakamadla. Supervision: Carlos Marcelo S. Figueredo. *Project administration*: Carlos Marcelo S. Figueredo. *Funding acquisition*: Not applicable. All authors have read and agreed to the published version of the manuscript.

## CONFLICTS OF INTEREST

The authors declare no conflicts of interest.

## Supporting information

Supporting information.

Supporting information.

Supporting information.

Supporting information.

Supporting information.

Supporting information.

## Data Availability

Data sharing is not applicable—no new data was generated, or the article describes entirely theoretical research.
