# Supplementary material for: A systematic review on neutrophils interactions with titanium and zirconia surfaces: Evidence from in vitro studies
Source: Clin Exp Dent Res. 2022 May 10;8(4):950–8. doi: 10.1002/cre2.582 (PMC9382042; doi:10.1002/cre2.582)
Supplement: Supplementary file 3 — Supporting information. [file CRE2-8-950-s004.docx]

**Supplementary material S1 - Database Search Strategies**

**PUBMED:** (Titanium OR zirconia) AND (neutrophils OR phagocyte OR neutrophils OR leukocyte OR granulocyte).

**EMBASE:** 'Titanium' OR 'zirconia' AND ('neutrophils' OR 'phagocyte' OR 'leukocyte' OR 'granulocyte')

**SCOPUS:** Titanium OR zirconia AND neutrophils OR phagocyte OR neutrophils OR leukocyte OR granulocyte.

**WEB OF SCIENCE:** Titanium OR zirconia AND neutrophils OR phagocyte OR neutrophils OR leukocyte OR granulocyte.
