# Supplementary figures and images for: A systematic review on neutrophils interactions with titanium and zirconia surfaces: Evidence from in vitro studies
Source: Clin Exp Dent Res. 2022 May 10;8(4):950–8. doi: 10.1002/cre2.582 (PMC9382042; doi:10.1002/cre2.582)

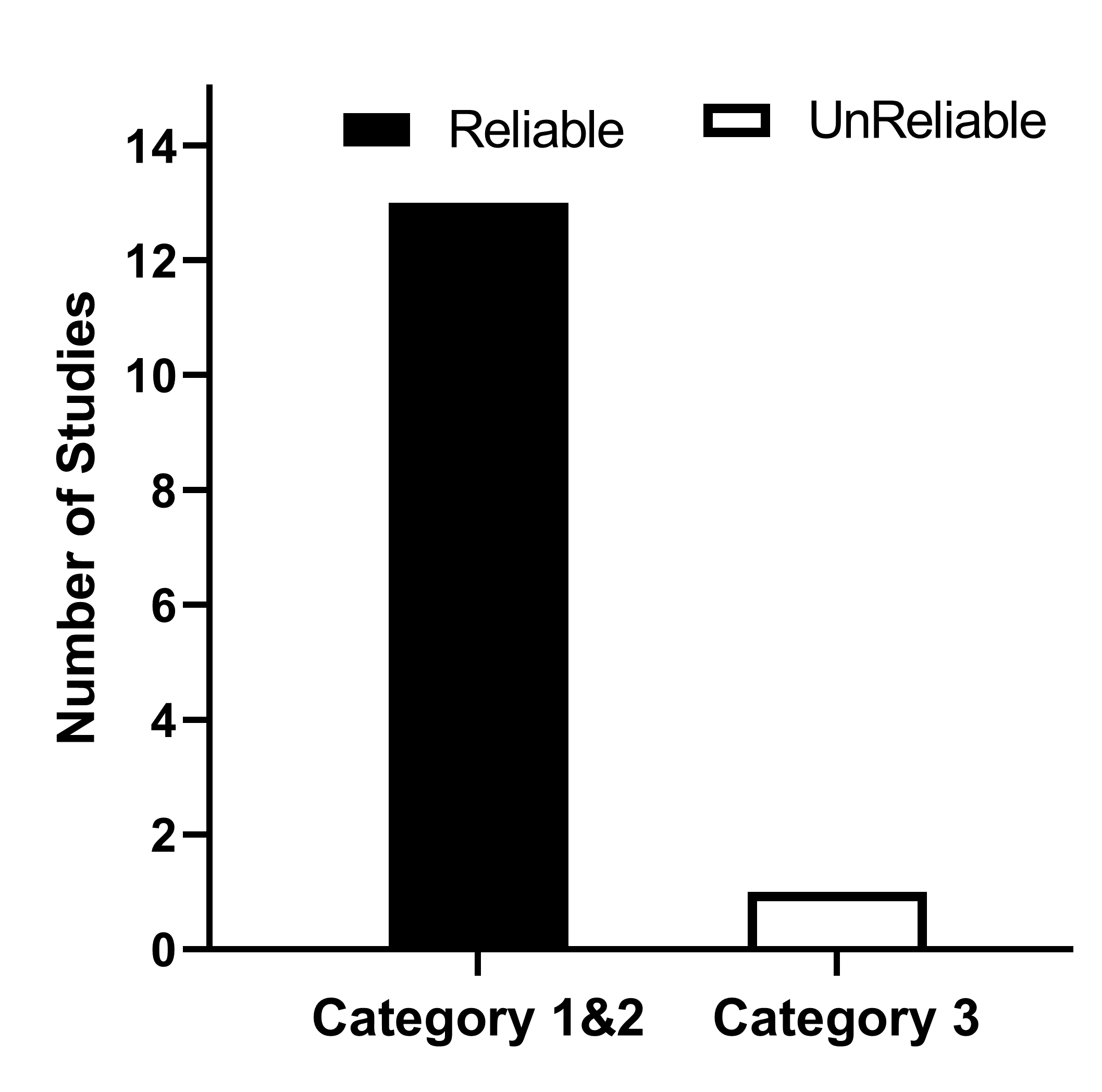

Supplement: Supplementary file 4 — Supporting information. [file CRE2-8-950-s005.tif]
