# Supplementary material for: A systematic review on neutrophils interactions with titanium and zirconia surfaces: Evidence from in vitro studies
Source: Clin Exp Dent Res. 2022 May 10;8(4):950–8. doi: 10.1002/cre2.582 (PMC9382042; doi:10.1002/cre2.582)
Supplement: Supplementary file 5 — Supporting information. [file CRE2-8-950-s003.docx]

**Supplementary Table S1 – Reasons for excluding after assessing eligibility criteria.**

| **ONLY ABSTRACTS/REVIEWS/CASE REPORTS** | | | |
| --- | --- | --- | --- |
|  | **AUTHOR** | **YEAR** | **TITLE** |
| 1 | Albertini, M. | 2015 | Advances in surfaces and osseointegration in implantology. Biomimetic surfaces |
| 2 | Radley, G. | 2017 | Assessment of leukocyte functionality in contact with a foreign surface under shear stress |
| 3 | Radley, G. | 2015 | Biomaterial-induced leukocyte activation in reference to ventricular assist devices |
| 4 | Gaydos, J. M. | 2000 | Bisphosphonate effect on neutrophil activation by titanium and hydroxyapatite implants |
| 5 | Asawang, K. | 2002 | Clinical instrumentation of titanium surfaces alters the neutrophil oxidative response. |
| 6 | Biguetti, C. C | 2016 | DAMPS/MYD88 axis in the modulation of host inflammatory and healing responses to classic titanium-based biomaterials in vivo |
| 7 | Tamura, K. | 2004 | Effects of Micro/Nano Particle Size on Cell Function and Morphology |
| 8 | Jorgenson, D. S. | 1996 | Have you seen this?': Leukocyte response to titanium implants |
| 9 | Rochford, E. T. J. | 2019 | Infection burden and immunological responses are equivalent for polymeric and metallic implant materials in vitro and in a murine model of fracture-related infection. |
| 10 | Smith, G. | 2015 | Infection in prosthetic material |
| 11 | Chung, L. | 2017 | Key players in the immune response to biomaterial scaffolds for regenerative medicine |
| 12 | Moore, M. A. | 2000 | Neutrophil response to titanium materials-micro compatibility following various surface treatments |
| 13 | Desai, J. | 2017 | Nano-and microparticles of different sizes and shapes induce neutrophil necroptosis and neutrophil extracellular traps. |
| 14 | Zimmerli, W. | 2011 | Pathogenesis of implant-associated infection: The role of the host |
| 15 | Podzimek, S. | 2016 | Proinflammatory cytokines production and metal hypersensitivity in patients with failed orthopaedic implants |
| 16 | Radley, G. | 2016 | The impact of artificial shear stress on leukocytes at a biomaterial interface |
| 17 | Dion, I. | 1992 | TiN coating: Physico-chemistry and leukocyte adhesion |
| 18 | Simonian, P. T. | 1995 | Titanium contamination of recycled Cell Saver blood in revision hip arthroplasty |
| **NO NEUTROPHILS/NO IMPLANT SURFACES/COATED SURFACES** | | | |
|  | **AUTHOR** | **YEAR** | **TITLE** |
| 1 | Rajan, A | 2018 | An in vitro study to evaluate and compare the hemocompatibility of titanium and zirconia implant materials after sandblasted and acid-etched surface treatment |
| 2 | Choi, J. | 2003 | Calcium phosphate coating of nickel-titanium shape memory alloys. Coating procedure and adherence of leukocytes and platelets |
| 3 | Ciapetti, G. | 1998 | Fluorescent microplate assay for respiratory burst of PMNs challenged in vitro with orthopaedic metals |
| 4 | Chen, F. S. | 1999 | In vitro and in vivo activation of polymorphonuclear leukocytes in response to particulate debris |
| 5 | Thewes, M | 2001 | Immunohistochemical characterization of the perivascular infiltrate in tissues adjacent to stainless steel implants compared with titanium implants. |
| 6 | Manivasagam, V. K. | 2020 | In Vitro Investigation of Hemocompatibility of Hydrothermally Treated Titanium and Titanium Alloy Surfaces |
| 7 | Burkhardt, M. A. | 2016 | Synergistic interactions of blood-borne immune cells, fibroblasts and extracellular matrix drive repair in an in vitro peri-implant wound healing model |
| 8 | Pascual, A. | 1992 | The effect of stainless steel, cobalt-chromium, titanium alloy, and titanium on the respiratory burst activity of human polymorphonuclear leukocytes powder |
| 9 | Contreras, R. | 2007 | Titanate biomaterials with enhanced anti-inflammatory properties |
| 10 | Tan, J. | 2002 | Topographical control of human neutrophil motility on micropatterned materials with various surface chemistry |
| 11 | Kubacki, G. W. | 2018 | The effect of the inflammatory species hypochlorous acid on the corrosion and surface damage of Ti-6Al-4V and CoCrMo alloys |
|  | **OTHER LANGUAGE** | | |
|  | **AUTHOR** | **YEAR** | **TITLE** |
| 1 | Plekhova, N. G. | 2011 | The metabolic activity of neutrophils and monocytes as the model for the study of the organism biocompatibility with different materials |
| 2 | Plekhova, N. G. | 2016 | Effect of modified coatings titanium implants on innate immunity cell |
