# Supplementary material for: A systematic review on neutrophils interactions with titanium and zirconia surfaces: Evidence from in vitro studies
Source: Clin Exp Dent Res. 2022 May 10;8(4):950–8. doi: 10.1002/cre2.582 (PMC9382042; doi:10.1002/cre2.582)
Supplement: Supplementary file 6 — Supporting information. [file CRE2-8-950-s002.docx]

**Table 2.** Risk of bias among the included studies

| **Study**  **Authors & Year** | ***Test Substance Identified*** | ***Purity of test substance*** | ***Source of substance*** | ***Nature of test substance*** | ***Test system described*** | ***Source of the test system*** | ***Information of test substance*** | ***Administration methods*** | ***Concentration*** | ***Timepoints*** | ***Negative controls*** | ***Positive controls*** | ***Replicates*** | ***Endpoints described*** | ***Endpoint investigated*** | ***Statistical investigation*** | ***Substance specific obtained*** | ***Results reliability*** | ***Total Score*** |
| --- | --- | --- | --- | --- | --- | --- | --- | --- | --- | --- | --- | --- | --- | --- | --- | --- | --- | --- | --- |
| *Kholy et al., 2020* | 1 | 1 | 1 | 1 | 1 | 1 | 1 | 1 | 1 | 1 | 1 | 1 | 1 | 1 | 1 | 1 | 1 | 1 | 18 |
| *Abaricia et al., 2020* | 1 | 1 | 1 | 1 | 1 | 1 | 1 | 1 | 1 | 1 | 1 | 1 | 1 | 1 | 1 | 1 | 1 | 1 | 18 |
| *Radley Et al., 2019* | 1 | 1 | 1 | 1 | 1 | 1 | 1 | 1 | 1 | 1 | 1 | 1 | 1 | 1 | 1 | 1 | 0 | 1 | 17 |
| *Radley et al.,2018* | 1 | 1 | 1 | 1 | 1 | 1 | 1 | 1 | 1 | 1 | 0 | 1 | 1 | 1 | 1 | 1 | 0 | 1 | 17 |
| *Vitkov et al., 2015* | 1 | 1 | 1 | 1 | 1 | 1 | 1 | 1 | 1 | 1 | 0 | 1 | 1 | 1 | 1 | 1 | 1 | 1 | 17 |
| *Campos et.al.,2014* | 1 | 1 | 1 | 1 | 1 | 1 | 1 | 1 | 1 | 1 | 0 | 1 | 1 | 1 | 1 | 1 | 1 | 1 | 17 |
| *Smith et al., 2013* | 1 | 0 | 0 | 0 | 0 | 1 | 1 | 1 | 1 | 1 | 0 | 1 | 0 | 1 | 1 | 1 | 0 | 1 | 11 |
| *Arvidsson et al., 2011* | 1 | 1 | 1 | 1 | 1 | 1 | 1 | 1 | 1 | 1 | 0 | 1 | 0 | 1 | 1 | 1 | 0 | 1 | 15 |
| *Schildhauer et al.,2009* | 1 | 1 | 1 | 1 | 1 | 1 | 1 | 1 | 1 | 1 | 1 | 1 | 1 | 1 | 1 | 1 | 1 | 1 | 18 |
| *Choi et al., 2003* | 1 | 1 | 1 | 1 | 1 | 1 | 1 | 1 | 1 | 1 | 1 | 1 | 1 | 1 | 1 | 1 | 1 | 1 | 18 |
| *Erikkson et al., 2001* | 1 | 1 | 1 | 0 | 1 | 1 | 1 | 1 | 1 | 1 | 0 | 0 | 0 | 1 | 1 | 1 | 1 | 1 | 14 |
| *Erikkson et al., 2001* | 1 | 1 | 1 | 1 | 1 | 1 | 1 | 1 | 1 | 1 | 1 | 1 | 1 | 1 | 1 | 1 | 1 | 1 | 18 |
| *Erikkson et al., 2001* | 1 | 1 | 1 | 1 | 1 | 1 | 1 | 1 | 1 | 1 | 0 | 1 | 1 | 1 | 1 | 1 | 0 | 1 | 17 |
| *Nygren et al., 1997* | 1 | 1 | 1 | 1 | 1 | 1 | 1 | 1 | 1 | 1 | 0 | 1 | 1 | 1 | 1 | 1 | 0 | 1 | 17 |
